# Cardiovascular Biomarkers in Nocturnal Hemodialysis and Their Association With Physical Performance

**DOI:** 10.1111/hdi.13265

**Published:** 2025-05-22

**Authors:** Manouk Dam, Laura M. M. de Haan, Tiny Hoekstra, Marc Vervloet, Frans J. van Ittersum, Peter J. M. Weijs, Brigit C. van Jaarsveld

**Affiliations:** ^1^ Department of Nutrition and Dietetics, Amsterdam Cardiovascular Sciences Amsterdam UMC, VU University Amsterdam Amsterdam the Netherlands; ^2^ Department of Nephrology Amsterdam UMC, VU University Amsterdam Amsterdam the Netherlands; ^3^ Department of Nephrology Radboud UMC Nijmegen the Netherlands; ^4^ Amsterdam University of Applied Sciences, Faculty of Sports and Nutrition Department of Nutrition and Dietetics the Netherlands; ^5^ Nephrocare Diapriva Dialysis Center Amsterdam the Netherlands

**Keywords:** cardiovascular biomarkers, nocturnal hemodialysis, physical performance

## Abstract

**Introduction:**

The cardiovascular biomarkers troponin T, N‐terminal pro‐B‐type natriuretic peptide, and fibroblast growth factor 23 are elevated in hemodialysis patients and associated with an increased cardiovascular mortality risk. Nocturnal hemodialysis improves the fluid status in hemodialysis patients. Therefore, we investigated whether nocturnal hemodialysis (7–8 h sessions) was associated with lower levels of troponin T, N‐terminal pro‐B‐type natriuretic peptide, and fibroblast growth factor 23 in comparison to conventional hemodialysis. Second, we investigated whether these biomarkers were independently associated with physical performance in hemodialysis patients.

**Methods:**

A prospective cohort of 33 hemodialysis patients was compared to 32 patients who voluntarily switched from conventional hemodialysis to nocturnal hemodialysis. First, we studied the difference between the two cohorts in change over 12 months of troponin T, N‐terminal pro‐B‐type natriuretic peptide, and fibroblast growth factor 23 with linear mixed models. Second, the associations between these biomarkers and physical‐activity monitor, six minute walk test, and physical component summary score were assessed at baseline, 6 and 12 months.

**Findings:**

N‐terminal pro‐B‐type natriuretic peptide increased 122% during conventional hemodialysis, whereas it decreased 31% during nocturnal hemodialysis (*p* = 0.001). In conventional hemodialysis, fibroblast growth factor 23 rose numerically by 19% (23%–66%) in 12 months, while a decline of 44% (21%–58%) was found in nocturnal hemodialysis patients (*p* = 0.17). Troponin T did not differ between groups. Regarding physical performance, a higher N‐terminal pro‐B‐type natriuretic peptide (per 1000 ng/L) and fibroblast growth factor 23 (per 1000 RU/mL) were associated with lower physical component summary scores of −0.02 (*p* = 0.02) and −0.04 (*p* = 0.05), respectively. Troponin T was not associated with physical performance.

**Discussion:**

Our findings showed that nocturnal hemodialysis was associated with a decrease in N‐terminal pro‐B‐type natriuretic peptide. This suggested that nocturnal hemodialysis diminished volume overload and thereby myocardial stretch. Additionally, lower levels of N‐terminal pro‐B‐type natriuretic peptide and fibroblast growth factor 23 were found to be associated with better self‐reported physical performance scores.

## 1 Introduction

Although dialysis treatment for patients with kidney failure is a lifesaving procedure, patients still experience a high disease burden. The majority of patients on hemodialysis have an increased risk of developing severe medical conditions, such as disturbances in mineral and bone health, left ventricular hypertrophy, cardiac calcifications, and myocardial stunning [1, 2, 3, 4], all contributing to elevated cardiovascular risk and high mortality [5]. Low levels of physical performance and exercise activities are seen among hemodialysis patients in comparison to healthy subjects [6, 7], which deteriorate cardiovascular health and survival in this population [8, 9].

Troponin T, N‐terminal pro‐B‐type natriuretic peptide, and fibroblast growth factor 23 are biomarkers associated with cardiovascular functioning. Troponin T is elevated after myocardial injury following ischemia and is moderately elevated in dialysis patients, probably as a consequence of mild myocardial ischemia during dialysis. The myocardial damage can lead to the progression of left ventricular hypertrophy [10] and left ventricular systolic dysfunction [11, 12, 13, 14]. N‐terminal pro‐B‐type natriuretic peptide is released in response to myocardial stretch following volume overload and is thus a reflection of the volume state [13, 15, 16]. Also, it is found to be elevated through myocardial ischemia and is a predictor of left ventricular systolic and diastolic dysfunction, left ventricular hypertrophy, cardiovascular events, later hospitalization for ischemic heart disease, and mortality [15, 17, 18, 19, 20]. Fibroblast growth factor 23 is involved in phosphate homeostasis through renal phosphorus handling, parathyroid hormone secretion, and vitamin D metabolism. Elevated fibroblast growth factor 23 levels in hemodialysis patients are attributable to reduced phosphate excretion and consequent parathyroid hormone secretion [21] and are associated with increased cardiovascular mortality, stroke, heart failure, and left ventricular hypertrophy [22, 23].

Over the years, different treatment modalities have been investigated as an attempt to improve clinical outcomes. Prolonged dialysis, often prescribed as nocturnal hemodialysis, showed promising results in this respect. Nocturnal hemodialysis is found to improve phosphate levels, hypertension, hyperparathyroidism, erythropoietin resistance, and to diminish ultrafiltration rates, hospitalization, and mortality, in comparison with conventional hemodialysis [24, 25, 26, 27, 28]. Only a few studies investigated whether cardiovascular biomarkers decreased during nocturnal hemodialysis and if this reflects clinical benefits, such as regression of left ventricular hypertrophy [29, 30, 31, 32]. We hypothesized that troponin T and N‐terminal pro‐B‐type natriuretic peptide could decrease by better volume control, less intradialytic hypotension, and so reduce myocardial strain in nocturnal hemodialysis patients. Fibroblast growth factor 23 could decrease as a result of better clearance of the hormone itself, or by improved phosphate control during longer hemodialysis sessions. Additionally, we speculated that these biomarkers might be associated with physical performance. Limited studies have investigated this topic in hemodialysis patients [33, 34, 35]. Therefore, the primary aim of this study was to investigate if nocturnal hemodialysis is associated with lower levels of troponin T, N‐terminal pro‐B‐type natriuretic peptide, and fibroblast growth factor 23 in comparison with conventional hemodialysis. The secondary aim was to investigate whether these cardiovascular biomarkers were associated with physical performance.

## 2 Materials and Methods

### 2.1 Study Design and Population

Data collection was part of the DiapriFIT study, a prospective, multicenter, observational cohort study comparing physical performance and protein‐energy wasting of nocturnal hemodialysis patients with conventional hemodialysis patients [36, 37]. Patients treated with conventional hemodialysis (three times, 3–4 h) were offered the option to switch to nocturnal hemodialysis (three times, 7–8 h), if this was medically possible according to their nephrologist. Exclusion criteria were dementia, unstable angina pectoris, recent myocardial infarction, severe pulmonary disease, life expectancy < 12 months, treatment incompliance, or planned renal transplantation [36]. The control group was patients who preferred conventional hemodialysis due to personal non‐medical preferences but were considered eligible for nocturnal hemodialysis. For nocturnal dialysis, a high‐flux Helixone^*R*^ filter was applied with 1.8 m^2^ surface area, blood flow of 180–220 mL/min, and dialysate flow of 500 mL/min. Further details regarding study design and sample size were published previously [36]. The study protocol was in accordance with the Declaration of Helsinki and approved by the Medical Ethics Committee of Amsterdam UMC (https://onderzoekmetmensen.nl/nl/trial/45059, accessed on February 20, 2024).

### 2.2 Outcome Parameters

#### 2.2.1 First Outcome—Cardiovascular Biomarkers

Troponin T, N‐terminal pro‐B‐type natriuretic peptide, and fibroblast growth factor 23 were measured before initiating nocturnal hemodialysis, after 6 and 12 months, to investigate the difference in change over time for the two cohorts. Blood samples were drawn following dialysis treatment and were stored at −80°C within 1 h. Serum samples of troponin T and N‐terminal pro‐B‐type natriuretic peptide levels were measured with Immunoassays by Roche Diagnostics. N‐terminal pro‐B‐type natriuretic peptide had an upper detection limit of 70,000 ng/L. Fibroblast growth factor 23 plasma samples (C‐terminal, RU/mL) were measured using an enzyme‐linked immunosorbent assay from Biomedica, Vienna, Austria, with a detection range of 220–16,700 RU/mL.

#### 2.2.2 Second Outcome—Physical Performance

Physical performance was measured with: (i) seven‐day physical activity monitor, (ii) six minute walk test, and (iii) physical component summary score measured with the kidney disease quality of life‐short form questionnaire, at baseline, 6 and 12 months. We selected these tests because they each address a different aspect of physical performance: physical daily activity (physical activity monitor), capacity and endurance (six minute walk test) and perceived physical health (physical component summary score). The physical activity monitor recorded daily activity for a week in minutes, and the average was used for the analyses. The six minute walk test measures walking distance during 6 min in meters (m) and was performed indoors on a 30 m long walking course [38]. The physical component summary score was derived from the kidney disease quality of life‐short form questionnaire (Dutch version 1.2) which contains domains on physical functioning, limitations caused by physical problems, pain, and general health. The physical component summary score is transformed in such a way that 50 represents the mean of the United States population with a standard deviation of 10 [39].

### 2.3 Statistical Analysis

Outcomes were reported as mean ± standard deviation (SD) or median (interquartile range, IQR) where appropriate. Linear mixed models were used with an intention‐to‐treat strategy to assess the relation between dialysis modality and biomarkers, with a random intercept. We hypothesized that there would be a difference between groups and a difference within groups over time; therefore, we added treatment, time, and an interaction of treatment*time as fixed variables into our model. In advance, we assumed that most outcomes would be linear, but we discussed that some outcomes might be most influenced during the first 6 months of nocturnal hemodialysis, after which a plateau effect could be reached (e.g., lower N‐terminal pro‐B‐type natriuretic peptide caused by better volume control in the first 6 months of nocturnal hemodialysis). Therefore, we analyzed time as a continuous variable and also as a categorical variable (baseline, 6 and 12 months) to investigate possible non‐linear developments as well. Gender, age, and duration of kidney replacement therapy were included as covariates in the model. Random slopes were considered, but these showed no improvement of the model. We present estimated marginal means for each biomarker in the corresponding figures instead of absolute biomarker levels since estimated marginal means take into account attrition and covariates.

In the analyses on physical performance, associations between each physical performance and each biomarker were analyzed. The regression coefficients were multiplied by 10 for troponin T and by 1000 for N‐terminal pro‐B‐type natriuretic peptide and fibroblast growth factor 23 for easier interpretation of the observed effects. For the relation of biomarkers and physical performance tests, data at all time points of both conventional and nocturnal hemodialysis patients were pooled, as treatment group nor time were relevant to the research question. Yet, these models did include treatment group and time (categorical variable) as confounders, in addition to the earlier mentioned confounders in the linear mixed model analysis. Statistical significance was set at *p* ≤ 0.05, and analyses were performed using IBM SPSS Statistics version 26.0.

## 3 Results

### 3.1 Participant Recruitment

**FIGURE 1:**
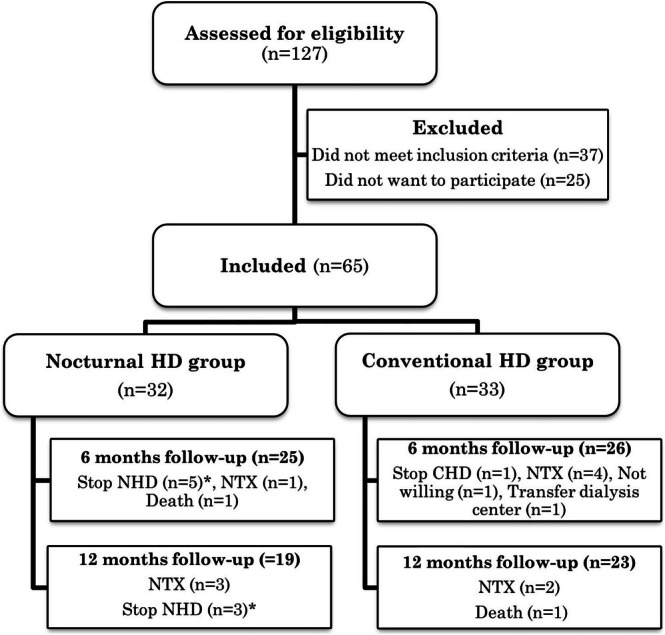
Participant flow diagram. *Mostly due to sleeping difficulties or transfer to home dialysis.

Assessed for eligibility were 127 patients, of which 65 met the inclusion criteria and provided informed consent. Of these, 32 patients switched from conventional hemodialysis to nocturnal hemodialysis, while 33 participants continued their conventional schedule. Due to several reasons, 6‐month data were available for 26 patients and 12‐month data for 22 patients in the conventional group and for 25 patients and 19 patients in the nocturnal group (Figure 1).

### 3.2 Baseline Characteristics and Dialysis Treatments

In the conventional hemodialysis group, 49% were male versus 56% in the nocturnal group (Table 1), with a mean age of 60.0 ± 14.0 and 50.0 ± 15.3 years, respectively. Diastolic blood pressure was 72 ± 12 mmHg in conventional and 79 ± 12 mmHg in nocturnal hemodialysis patients, and kidney replacement therapy vintage was 40 (15–123) and 21 (9–75) months respectively. Treatment characteristics and clinical parameters at baseline and during follow‐up are presented in Table 2. As expected, dialysis dose increased in nocturnal hemodialysis (conventional 2.33 ± 0.42 and nocturnal 2.80 ± 0.21, at 12 months) and post‐dialysis urea values declined more in the nocturnal hemodialysis group. Both systolic and diastolic blood pressure remained stable in conventional and decreased in nocturnal hemodialysis, with a mean of 6% to 8%, respectively. Additionally, phosphate and parathyroid hormone increased in conventional and declined in nocturnal hemodialysis after 12 months.

**TABLE 1:** Patient characteristics of the study population at baseline.

Characteristics	Conventional hemodialysis (*n* = 33)	Nocturnal hemodialysis (*n* = 32)	*p*
Gender (male)	16 (49%)	18 (56%)	0.4
Age (years)	60.0 ± 14.0	50.0 ± 15.3	0.01
Cause of end‐stage kidney disease (%)			—
Diabetes	8 (24%)	5 (16%)	
Hypertension	6 (18%)	5 (16%)	
Polycystic disease	1 (3%)	3 (9%)	
Other	18 (55%)	19 (59%)	
History of cardiovascular disease (%)			
Coronary artery disease	7 (21%)	4 (13%)	0.3
Heart failure	4 (12%)	2 (6%)	0.4
Hypertension	21 (64%)	25 (78%)	0.2
Hyperlipidemia	10 (30%)	9 (28%)	0.8
Neoplasms	3 (9%)	7 (20%)	0.2
Rheumatic diseases	2 (6%)	1 (3%)	0.6
Medications			
Angiotensin‐converting enzyme inhibitors	8 (24%)	7 (22%)	0.8
Angiotensin II receptor blockers	2 (6%)	2 (6%)	0.9
Diuretics	15 (46%)	18 (56%)	0.4
Statins	13 (39%)	8 (25%)	0.2
Antihypertensives	17 (52%)	19 (59%)	0.5
Charlson Comorbidity Index			0.5
2 (only end‐stage kidney disease)	7 (21%)	11 (34%)	
3–4 (intermediate comorbidity)	14 (42%)	11 (34%)	
≥ 5 (high comorbidity)	12 (36%)	10 (31%)	
Systolic blood pressure^a^ (mmHg)	134 ± 25	141 ± 22	0.3
Diastolic blood pressure^a^ (mmHg)	72 ± 12	79 ± 12	0.02
Body weight (kg)	77.3 ± 18.7	81.9 ± 20.3	0.4
Body mass index (kg/m^2^)	26.9 ± 6.5	28.0 ± 6.7	0.5
Duration of kidney replacement therapy in months, prior to start of study	40 (15–123)	21 (9–75)	0.1
Anuria, *n* (%)	16 (49%)	10 (31%)	0.3

*Note*: Categorical data represented as frequency (percentage), continuous data as mean ± standard deviation or as median with interquartile range. Independent *t*‐tests were used for normally distributed variables and Mann–Whitney *U*‐tests for skewed data. Chi‐squared tests were used for categorical variables.

^a^ Blood pressure measurements were taken after hemodialysis treatment, means of 2 weeks of hemodialysis sessions.

**TABLE 2:** Characteristics of dialysis regimens and changes in clinical parameters between conventional and nocturnal hemodialysis during 12 months.

	Conventional hemodialysis^a^	Nocturnal hemodialysis^a^	*p*
Dialysis frequency, times/week			
Baseline	2.8 ± 0.5	2.9 ± 0.3	0.3
6 months	2.9 ± 0.5	2.9 ± 0.3	1.0
12 months	2.9 ± 0.4	2.8 ± 0.4	0.8
Dialysis duration per session, hours			
Baseline	3.9 ± 0.4	3.9 ± 0.3	0.5
6 months	3.9 ± 0.4	8.0 ± 0.2	< 0.001
12 months	3.9 ± 0.5	7.9 ± 0.3	< 0.001
Dialysis dose (standardized Kt/V)			
Baseline	2.08 ± 0.45	2.11 ± 0.36	0.8
6 months	2.30 ± 0.37	2.66 ± 0.33	< 0.001
12 months	2.33 ± 0.42	2.80 ± 0.21	< 0.001
Systolic blood pressure^b^ (mmHg)			
Baseline	134 ± 25	141 ± 22	0.3
6 months	133 ± 27	137 ± 22	0.5
12 months	134 ± 28	132 ± 25	0.8
Diastolic blood pressure^b^ (mmHg)			
Baseline	72 ± 12	79 ± 12	0.02
6 months	69 ± 13	77 ± 13	0.05
12 months	71 ± 14	73 ± 15	0.6
Pre‐dialysis urea (mmol/L)			
Baseline	26.33 ± 6.33	24.07 ± 6.46	0.2
6 months	26.43 ± 5.46	24.06 ± 4.76	0.1
12 months	25.21 ± 6.43	24.26 ± 4.03	0.6
Post‐dialysis urea (mmol/L)			
Baseline	8.69 ± 3.21	9.44 ± 3.99	0.4
6 months	7.88 ± 2.89	6.00 ± 2.65	0.02
12 months	7.59 ± 3.08	5.22 ± 1.43	0.004
Phosphate (mmol/L)			
Baseline	1.63 ± 0.38	1.72 ± 0.56	0.5
6 months	1.59 ± 0.33	1.60 ± 0.69	0.3
12 months	1.84 ± 1.27	1.54 ± 0.60	0.9
Albumin (g/L)			
Baseline	40.2 ± 3.6	40.5 ± 2.2	0.7
6 months	39.5 ± 3.7	41.4 ± 1.8	0.03
12 months	39.9 ± 3.7	40.8 ± 2.4	0.3
Hemoglobin (mmol/L)			
Baseline	7.1 ± 0.6	7.1 ± 0.9	0.8
6 months	7.0 ± 0.8	7.2 ± 0.7	0.4
12 months	6.6 ± 0.9	7.2 ± 0.5	0.02
Parathyroid hormone (pmol/L)			
Baseline	37 (23–53)	37 (19–80)	0.6
6 months	33 (24–58)	37 (20–74)	0.7
12 months	42 (25–51)	31 (19–41)	0.2

^a^ Values are reported as means with standard deviation or median with interquartile range when appropriate; baseline values were both cohorts following a conventional dialysis treatment. Independent *t*‐tests were used for normally distributed variables and Mann–Whitney *U*‐tests for skewed data. Group sizes varied at time points. At baseline *n*_conventional hemodialysis_ = 33, *n*_nocturnal hemodialysis_ = 32; at 6 months *n*_conventional hemodialysis_ = 26, *n*_nocturnal hemodialysis_ = 25; and at 12 months *n*_conventional hemodialysis_ = 22, *n*_nocturnal hemodialysis_ = 19.

^b^ Blood pressures were measured after hemodialysis, and each measurement represents mean blood pressures during 2 weeks of hemodialysis treatment.

### 3.3 Cardiovascular Biomarkers in Nocturnal Hemodialysis

**FIGURE 2:**
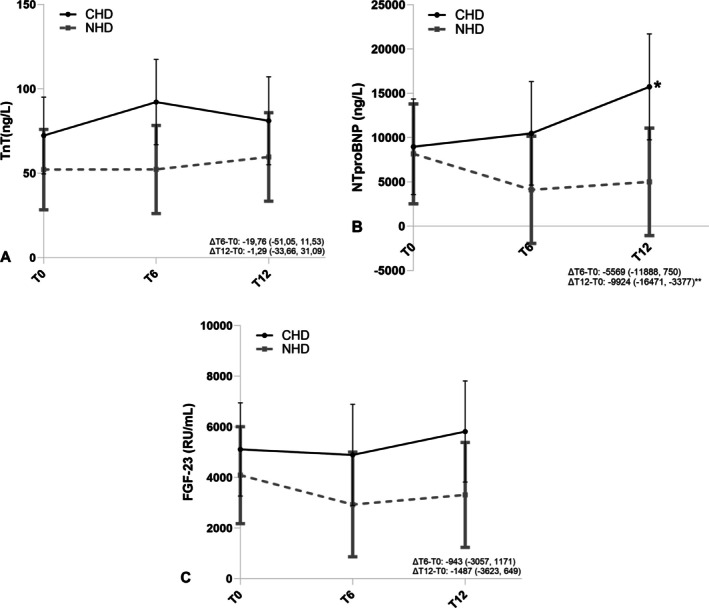
Within and between group differences of the biomarkers between nocturnal hemodialysis and conventional hemodialysis at baseline (T0), 6 months (T6) and 12 months (T12) of treatment on (A) troponin T, (B) N‐terminal pro‐B‐type natriuretic peptide and (C) fibroblast growth factor 23. Figures display the difference in 6 months (∆T6 − T0) and 12 months (∆T12 − T0) change between nocturnal hemodialysis and conventional hemodialysis. Figures are based on estimated marginal means and the corresponding 95% confidence intervals of a linear mixed model including covariates. *p = 0.004, mean in conventional hemodialysis cohort after 12 months differs significantly from baseline **p = 0.012, change in mean after 12 months differs significantly in nocturnal hemodialysis compared to the conventional hemodialysis group.

Troponin T median levels were somewhat lower at baseline in the nocturnal compared to the conventional hemodialysis group; troponin T increased 45% in conventional and 75% in nocturnal hemodialysis, but were still lower at 12 months in nocturnal compared to conventional hemodialysis (Table 3). No significant differences were found in troponin T after 1 year of nocturnal hemodialysis compared to conventional hemodialysis (−1.3, 95% CI −33.6 to 31.1, *p* = 0.94). Median N‐terminal pro‐B‐type natriuretic peptide levels increased 122% in conventional and decreased 31% in nocturnal hemodialysis over 12 months. There was a significant treatment effect of nocturnal hemodialysis treatment (−9924, 95% CI −16,471 to −3377, *p* < 0.001). The median level of fibroblast growth factor 23 increased 19% in conventional while in nocturnal hemodialysis it reduced by 44%. In linear mixed‐models, the treatment effect after 1 year of nocturnal compared to conventional hemodialysis was −1487, 95% CI −3623 to 649, *p* = 0.17. In Figure 2, the estimated marginal means of all biomarkers are presented.

**TABLE 3:** Levels of cardiovascular biomarkers during 12 months of nocturnal hemodialysis compared to conventional hemodialysis.

	Levels of cardiovascular biomarkers (median—interquartile range)			Linear mixed model of 12 months treatment effect of nocturnal hemodialysis compared to conventional hemodialysis^a^	
	Baseline^b^	6 months^b^	12 months^b^	Difference (95% CI)	*p*
Troponin T (ng/L)				−1.3 (−33.6 to 31.1)	0.94
CHD	50.0 (36.3–95.8)	66.0 (32.8–113.0)	72.5 (44.8–114.3)		
NHD	26.5 (21.0–56.8)	27.0 (21.0–47.5)	46.5 (18.0–85.3)		
N‐terminal pro‐B‐type natriuretic peptide (ng/L)				−9924 (−16,471 to −3377)	0.00
CHD	2793 (1185–7360)	3720 (1421–11,737)	6179 (2154–30,772)		
NHD	3661 (776–7533)	1512 (722–4275)	2543 (995–3805)		
Fibroblast growth factor 23 (RU/mL)				−1487 (−3623 to 649)	0.17
CHD	2603 (960–8101)	2445 (1127–8557)	3091 (1177–13,473)		
NHD	2670 (703–5390)	1452 (839–2253)	1486 (553–2280)		

^a^ A linear mixed model analysis was conducted with a random intercept and fixed factors included treatment, time, and treatment × time interaction. Covariates gender, age, and kidney replacement vintage were included in all models. Difference (95% CI) and *p* values are presented for the comparison between nocturnal hemodialysis and conventional hemodialysis during 12 months of treatment.

^b^ Group sizes varied during follow‐up: at baseline *n*_conventional hemodialysis_ = 33, *n*_nocturnal hemodialysis_ = 32, at 6 months *n*_conventional hemodialysis_ = 26, *n*_nocturnal hemodialysis_ = 25, and at 12 months *n*_conventional hemodialysis_ = 22, *n*_nocturnal hemodialysis_ = 19.

### 3.4 Cardiovascular Biomarkers and Physical Performance

Previously, we found that nocturnal hemodialysis patients improved their walking distance (6 min walk test) and that physical component summary scores and nutritional status improved after 1 year of nocturnal hemodialysis [37]. In the present study, we investigated the associations of each biomarker with physical performance tests.

The physical activity monitor showed a mean baseline value of 72 ± 55 min/day in the complete patient group. After 1 year, this activity decreased to 60 ± 41 min/day. No associations were found among the cardiovascular biomarkers and the physical activity monitor (troponin T *p* = 0.32; N‐terminal pro‐B‐type natriuretic peptide *p* = 0.34; fibroblast growth factor 23 *p* = 0.73, Table 4).

**TABLE 4:** Associations of cardiovascular biomarkers and parameters of physical performance in all patients.

	Crude model^a^		Model with adjustment for confounders^a^	
	Effect size (95% CI)^b^	*p*	Effect size (95% CI)^b^	*p*
Physical activity monitor (min/day)				
Troponin T (per 10 ng/L)	−10.2 (−2.6 to 0.6)	0.20	−0.8 (−2.4 to 0.8)	0.32
N‐terminal pro‐B‐type natriuretic peptide (per 1000 ng/L)	−0.4 (−1.0 to 0.2)	0.16	−0.3 (−0.8 to 0.3)	0.34
Fibroblast growth factor 23 (per 1000 RU/ml)	−0.8 (−2.8 to 1.3)	0.45	−0.4 (−2.4 to 1.7)	0.73
6 min walk test (meters)				
Troponin T (per 10 ng/L)	−0.3 (−3.8 to 3.2)	0.86	−0.8 (−4.1 to 2.5)	0.65
N‐terminal pro‐B‐type natriuretic peptide (per 1000 ng/L)	−0.9 (−2.9 to 1.0)	0.34	−0.5 (−2.3 to 1.2)	0.53
Fibroblast growth factor 23 (per 1000 RU/ml)	−3.3 (−8.1 to 1.4)	0.17	−2.5 (−6.6 to 1.5)	0.22
Physical component summary score				
Troponin T (per 10 ng/L)	−0.2 (−0.5 to 0.1)	0.19	−0.2 (−0.5 to 0.1)	0.25
N‐terminal pro‐B‐type natriuretic peptide (per 1000 ng/L)	−0.2 (−0.3 to −0.03)	0.02	−0.2 (−0.3 to −0.02)	0.02
Fibroblast growth factor 23 (per 1000 RU/mL)	−0.5 (−0.9 to −0.1)	0.02	−0.4 (−0.8 to −0.003)	0.05

^a^ Data were pooled from both cohorts at all‐time points. A linear mixed model analysis was conducted with a random intercept to adjust for the dependency of the repeated measurements within patients. Fixed factors consisted of the biomarker and covariates gender, age, history of kidney replacement therapy, treatment group, and time point. Allocation to either treatment group or time point of measurement was only included in the model as covariates. The presented *p* values and 95% confidence intervals (CI) refer to the fixed effect of the biomarker on the physical performance measurement.

^b^ The effect sizes and 95% CI are displayed as a multiple of 1000 for fibroblast growth factor 23 and N‐terminal pro‐B‐type natriuretic peptide and as a multiple of 10 for Troponin T, to allow for better interpretation of the effect sizes considering the observed levels of the biomarkers.

The 6 min walk test showed rather stable results during 1 year, with mean values of 348 ± 131 m at baseline and 345 ± 149 m at 12 months. No associations were found among troponin T, N‐terminal pro‐B‐type natriuretic peptide, and fibroblast growth factor 23 with the 6 min walk test.

Mean physical component summary scores started at 37 ± 12 points at baseline and were 38 ± 13 after 1 year. No associations were found with troponin T, but N‐terminal pro‐B‐type natriuretic peptide showed a significant association with physical component summary scores, such that a 1000 ng/L increase in N‐terminal pro‐B‐type natriuretic peptide was associated with a decrease of −0.2 in physical component summary score over 1 year (95% CI −0.3 to −0.02, *p* = 0.02). Similar results were seen for fibroblast growth factor 23: a 1000 RU/mL rise was associated with a decrease of −0.4 in physical component summary score in 1 year (95% CI −0.8 to −0.003, *p* = 0.05).

## 4 Discussion

This study investigated the effect of nocturnal hemodialysis compared to conventional hemodialysis treatment on the cardiac biomarkers troponin T, N‐terminal pro‐B‐type natriuretic peptide, and fibroblast growth factor 23. Lower levels of N‐terminal pro‐B‐type natriuretic peptide were found at 6 and 12 months of nocturnal hemodialysis treatment, compared to conventional hemodialysis. For troponin T levels and fibroblast growth factor 23 values, no statistically significant differences were found. Second, we investigated a possible association of the biomarkers with physical performance tests and found that higher levels of N‐terminal pro‐B‐type natriuretic peptide and fibroblast growth factor 23 were associated with lower physical component summary scores.

At 6 and 12 months of nocturnal hemodialysis treatment, we found lower levels of N‐terminal pro‐B‐type natriuretic peptide compared to the conventional hemodialysis group. N‐terminal pro‐B‐type natriuretic peptide is a marker for increased myocardial stretch following volume overload [13, 15, 16]. We assume that lower levels in nocturnal hemodialysis patients are induced by improved volume control and reduced myocardial strain due to lower afterload. This reflects in better regulation of blood pressure, as was seen in our nocturnal hemodialysis group, which suggests that our hypothesis is plausible. Ortega et al. found similar results in a longitudinal study on a strict volume control strategy among 46 conventional hemodialysis patients. They found an overall better control of systolic blood pressure, stable N‐terminal pro‐B‐type natriuretic peptide levels and in patients in the highest quartile of N‐terminal pro‐B‐type natriuretic peptide levels a reduction of N‐terminal pro‐B‐type natriuretic peptide values after 12 months [15]. This demonstrates that strict volume control and regulation of blood pressure maintain or reduce N‐terminal pro‐B‐type natriuretic peptide levels and may lower the risk for heart failure. To our knowledge, only a few studies investigated this topic in nocturnal hemodialysis patients, with conflicting results. Jefferies et al. showed trends on decreased values of N‐terminal pro‐B‐type natriuretic peptide and less dialysis‐induced myocardial stunning in nocturnal hemodialysis, in comparison to conventional hemodialysis patients, in a cross‐sectional study [32]. However, in a Canadian cohort with 37 nocturnal hemodialysis patients, changes in N‐terminal pro‐B‐type natriuretic peptide levels were not found to be correlated with left atrial volume or ejection fraction [40, 41]. It is possible that the improvements after conversion to nocturnal hemodialysis can only be confirmed in cardiac changes in larger patient groups.

In this study, we found no differences in troponin T levels between treatment groups. Mildly elevated troponin T values in chronic kidney disease patients could be caused by silent myocardial ischemia or left ventricular hypertrophy [12]. We hypothesized, as with N‐terminal pro‐B‐type natriuretic peptide, that less myocardial strain occurs due to lower afterload and a lower dialysis ultrafiltration rate. However, despite better control of volume seen in previous nocturnal hemodialysis studies [24, 42] and the lower blood pressure seen in our patients, no differences in troponin T levels were shown. Our results are in line with the study of Jefferies et al. where no significant differences in troponin T levels were found between nocturnal hemodialysis and conventional hemodialysis patients, although interesting reductions in their home‐based dialysis groups were observed. They mentioned low power as a limitation in their study to remark relevant changes of biomarkers, which is also a limitation in our study [32]. Besides, our nocturnal hemodialysis group started with low median troponin T levels of 26.5 ng/L. The conventional hemodialysis group started with a median level of 50 ng/L, which seems more in accordance with other studies on hemodialysis [12, 15]. This could suggest that our nocturnal hemodialysis population had less cardiac ischemia at start, which can also be deduced from somewhat lower age and a shorter vintage of kidney replacement therapy. By these differences between groups at baseline, it is less likely to detect a decline that had a lower value at baseline, even with our multilinear regression analyses, as troponin T values were relatively good at start.

A declining trend of median fibroblast growth factor 23 levels was observed in the nocturnal hemodialysis group, which was not statistically significant. Additionally, an increase in the conventional hemodialysis group was shown. We originally speculated that fibroblast growth factor 23 might decrease during nocturnal hemodialysis, as a consequence of enhanced clearance due to longer dialysis sessions. However, since nocturnal hemodialysis is found to reduce serum phosphate levels, it is also reasonable to speculate that fibroblast growth factor 23 would decrease by better phosphate clearance and parathyroid hormone regulation. We think it is probably multifactorial, since it becomes clearer that the regulation of fibroblast growth factor 23 is influenced by a scale of potential underlying mechanisms [43]. A few other studies investigated the relation of fibroblast growth factor 23 with nocturnal hemodialysis and/or cardiovascular outcomes, but results were conflicting. Similar results as in our study were seen in a post hoc analysis from the Frequent Hemodialysis Network trial, where reduced levels of fibroblast growth factor 23 were observed in the nocturnal group, but this was not statistically different between groups [31]. In a retrospective study of Kang et al., fibroblast growth factor 23 values were investigated in nocturnal hemodialysis in relation to left ventricular volume index and survival. Significant lower values were presented in nocturnal hemodialysis patients as compared to conventional hemodialysis patients at 1 year of follow‐up, and this was associated with diminished left ventricular hypertrophy [30]. Two other studies investigated fibroblast growth factor 23 levels in relation to left ventricular mass index [29] and epicardial and paracardial adipose tissue [40], but found no differences or associations with fibroblast growth factor 23. Whether nocturnal hemodialysis lowers fibroblast growth factor 23 values in the long term and if this would result in less cardiovascular morbidity and mortality remains to be elucidated.

In this study, we found an association of increasing N‐terminal pro‐B‐type natriuretic peptide with lower self‐reported physical component summary scores, but this was not found for the objective measures of the physical activity monitor or 6 min walk test. We hypothesized that lower levels of N‐terminal pro‐B‐type natriuretic peptide are a reflection of a better control of volume and less cardiac wall stress and thus influence physical performance positively. It is possible that patients feel better with lower N‐terminal pro‐B‐type natriuretic peptide values, but do not directly change their daily activity pattern or start exercising, which could explain the association between higher N‐terminal pro‐B‐type natriuretic peptide and lower self‐reported physical component summary scores but not with the physical activity monitor or 6 min walk test. Our results regarding physical component summary scores were not in line with the study of Williams et al., who found no relation of N‐terminal pro‐B‐type natriuretic peptide levels and the physical domains of health‐related quality of life in hemodialysis patients [33]. The study of Smith et al. investigated N‐terminal pro‐B‐type natriuretic peptide with several physical performance tests, where N‐terminal pro‐B‐type natriuretic peptide was associated with a decrease in baseline scores of the 6 min walk test, but no association was found with changes in the physical performance tests over 24 months [44].

Comparable results were seen in our data with fibroblast growth factor 23 values, where higher fibroblast growth factor 23 values were associated with lower physical component summary scores. A possible explanation could be that elevated fibroblast growth factor 23 impairs physical performance, but the relation could also be the other way around: better physical performance might influence fibroblast growth factor 23. This latter causal relation was suggested in a recent meta‐analysis of four randomized controlled trials, in which both endurance and aerobic exercises decreased fibroblast growth factor 23 [45].

Whether these lower physical component summary scores as seen with higher fibroblast growth factor 23 and N‐terminal pro‐B‐type natriuretic peptide are clinically relevant depends on the individual values of N‐terminal pro‐B‐type natriuretic peptide and fibroblast growth factor 23. A difference of 3 points has been suggested as clinically relevant [39], so a clinical difference might only be experienced in patients who have higher increased values of these biomarkers.

In this study, we found no associations of troponin T with the physical performance tests. Limited evidence is available from studies investigating troponin T in relation to physical performance in hemodialysis patients. Williams et al. found in a cohort of 596 patients that high troponin T levels were independently associated with deterioration of physical function during 96 weeks of hemodialysis [33]. It is possible that we could not duplicate this finding due to limited power.

This study has strengths and limitations. The strength of this study is that we investigated two different dialysis modalities in two comparable groups. The major limitation of this study is that patients were not randomized to either modality, making residual selection bias by unnoticed variables possible. Also, since the relatively small groups, it was not possible to apply pseudo‐randomization with propensity scores. Indeed, several baseline characteristics that were captured were not equal between groups, since the nocturnal hemodialysis group had a significantly lower age and shorter history of kidney replacement therapy. This could explain part of the variation of biomarkers at baseline. Therefore, we adjusted for age, gender, and kidney replacement therapy vintage in all analyses and assessed the changes and not the absolute values of the biomarkers, but the possibility of residual confounding cannot be excluded. Another limitation is that the blood samples were drawn after dialysis treatment and therefore could be a short‐term effect of more clearance during nocturnal hemodialysis treatment. This could be possible for troponin T and N‐terminal pro‐B‐type natriuretic peptide, although some studies show increases post dialysis treatment as well [19, 46, 47]. There is almost no clearance of fibroblast growth factor 23 during hemodialysis treatment [48, 49]. Blood samples needed to be processed directly (within a maximum of 1 h), which could not always be guaranteed during nighttime by the laboratory, so we chose to draw samples after dialysis treatment. Nevertheless, we found it plausible that if nocturnal hemodialysis would influence the biomarkers, that the average values during the week would be lower and we could still detect changes over time. Furthermore, we pooled all data from the two cohorts to analyze our second outcome because we found the sample size of the cohorts too small to investigate this outcome longitudinally.

In conclusion, the high cardiovascular burden in hemodialysis patients remains an important target for therapeutic strategy development. Our findings indicate that nocturnal hemodialysis lowers N‐terminal pro‐B‐type natriuretic peptide. This suggests that nocturnal hemodialysis alleviates the risk of volume overload and myocardial stretch. Additionally, lower levels of N‐terminal pro‐B‐type natriuretic peptide and fibroblast growth factor 23 are associated with better perceived physical performance.

## Conflicts of Interest

The authors declare no conflicts of interest.

## Data Availability

The data that support the findings of this study are available on request from the corresponding author. The data are not publicly available due to privacy or ethical restrictions.
